# Roles of Reactive Oxygen Species in Relationships Between Viral Infections and Alzheimer’s Disease and Related Dementia

**DOI:** 10.3390/antiox15010066

**Published:** 2026-01-03

**Authors:** Gunel Ayyubova, Fariha E. Bablu, Nazrin Rahimli, Leyla Aghayeva, Elijah M. Springer, Fada A. Alghenaim, Yuichiro J. Suzuki

**Affiliations:** 1 Department of Cytology, Embryology and Histology, Azerbaijan Medical University, Baku AZ1022, Azerbaijan; 2 Department of Pharmacology and Physiology, Georgetown University Medical Center, Washington, DC 20057, USA; 3 Department of Pharmacology and Toxicology, College of Pharmacy, Qassim University, Buraydah 52571, Saudi Arabia

**Keywords:** Alzheimer’s disease, COVID-19, dementia, free radicals, herpes virus, neurological disease, reactive oxygen species, spike protein, virus

## Abstract

Emerging evidence suggests that viral infections may contribute to the onset and progression of Alzheimer’s disease (AD) and other forms of dementia. Understanding the mechanism of viral involvement in the pathogenesis of AD and related dementia (ADRD) could contribute to reducing the burden caused by these conditions, which affect a large portion of the aging population. Some studies indicate the link between AD and viral infections, notably coronaviruses and herpesviruses. In AD, excessive production of reactive oxygen species (ROS) results in the modifications of lipids, proteins, and nucleic acids, contributing to synaptic dysfunction and cognitive impairments. Experimental evidence suggests that viral infections linked to ADRD induce the cellular production of ROS, possibly contributing to the pathogenesis of these conditions. Despite significant advances in defining the roles of ROS in neurological disorders and viral infections, the specific roles of ROS in virus-associated ADRD have not been thoroughly investigated. The main objective of this review article is to comprehensively provide information on the experimental evidence for the production of ROS by viruses to help the readers investigate the role of ROS in the relationship between viral infections with ADRD.

## 1. Introduction

Infections caused by certain viruses have been implicated in the pathogenesis of various complications, including Alzheimer’s disease (AD) and other forms of dementia [1,2,3]. The relationships between viruses, redox status, and neurological disorders like AD have been discussed in previous reviews [4,5,6]. However, the role of reactive oxygen species (ROS) in the link between viral infections and AD and related dementia (ADRD) remains controversial and unclear [7].

**Figure 1:**
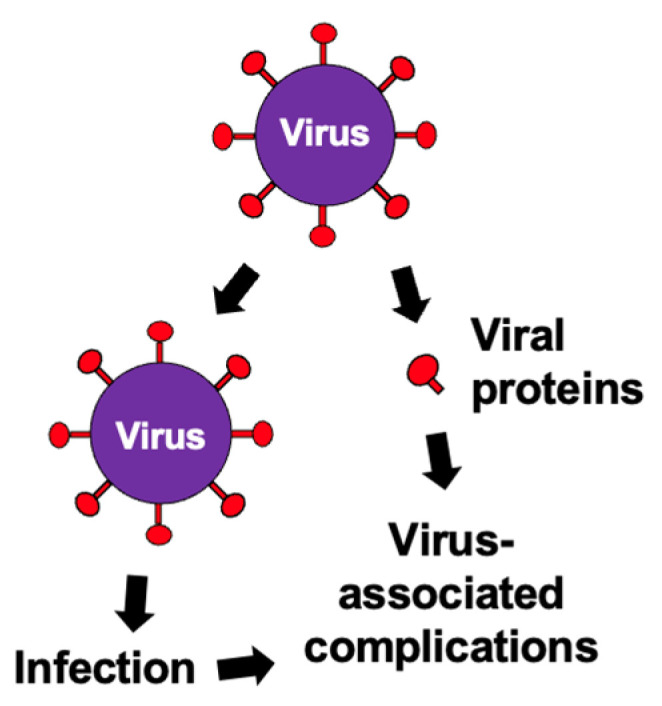
Scheme depicting viral infection and virus-associated complications. Virus-mediated complications can arise from the direct effects of viral infection, including the death of host cells. Additionally, freely circulating viral proteins released from the virus contribute to the promotion of these virus-mediated complications.

Viruses typically enter host cells by interacting with cell surface receptors via membrane fusion proteins. Virus-mediated complications can arise from the direct effects of viral infection, including alterations to host cells, such as cell death. Additionally, the recent coronavirus disease 2019 (COVID-19) pandemic, caused by severe acute respiratory syndrome coronavirus 2 (SARS-CoV-2), has highlighted the potential pathological role of freely circulating viral fusion proteins such as the spike protein in the blood of patients experiencing virus-mediated complications [8,9]. These two mechanisms contributing to virus-mediated pathology are depicted in the scheme shown in Figure 1.

**Figure 2:**
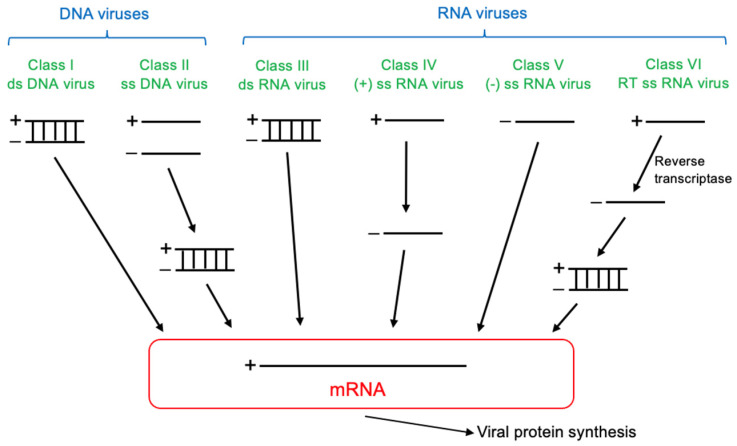
Classification of Viruses. According to the Baltimore Classification System, viruses are categorized into DNA viruses and RNA viruses. DNA viruses include both double-stranded (ds) and single-stranded (ss) variants. RNA viruses encompass double-stranded RNA viruses, positive-sense (+) single-stranded RNA viruses, negative-sense (−) RNA viruses, and reverse-transcribing (RT) viruses such as the human immunodeficiency virus. RT, reverse transcribing [12,13].

Viruses are microscopic, obligate intracellular parasites that require living host cells for replication. Despite the diversity in the diseases they cause and the tissues they affect, all viruses share a fundamental structure composed of nucleic acids (e.g., DNA or RNA) and proteins [10]. These viral genomes are encased in a protective protein shell called the capsid, which shields them from environmental degradation. Some viruses also possess a lipid envelope embedded with glycoproteins that facilitate host cell recognition and entry. The nucleic acid and capsid together form the nucleocapsid, the basic structural unit of the virion. This simplicity enables viruses to efficiently exploit host systems, contributing to their adaptability and pathogenicity. However, their lack of cellular organelles renders them entirely dependent on the host’s metabolic machinery for replication, defining them as genetic parasites. Viruses are classified based on key features such as virion size, capsid structure, type and nature of nucleic acid, physical properties, host range, and associated diseases [10,11]. According to the Baltimore Classification System [12,13], viruses are divided into DNA and RNA viruses (Figure 2). DNA viruses include both double-stranded and single-stranded variants. RNA viruses encompass double-stranded RNA viruses, positive-sense single-stranded RNA viruses, negative-sense RNA viruses, and reverse-transcribing viruses.

Viruses that can infect the nervous system, referred to as neurotropic viruses, employ specialized strategies to target and exploit neural tissues [14]. Moreover, a recent report by Duff et al. [15] provided evidence that SARS-CoV-2 infection can lead to increased AD-like pathology.

In AD, the excessive production of ROS leads to oxidative stress, which contributes to synaptic dysfunction and cognitive impairments. Viral infections linked to ADRD may induce the cellular production of ROS, contributing to the pathogenesis of these neurological conditions. The main objective of this review article is to comprehensively compile the experimental evidence that demonstrates the production of ROS by viruses and viral components to help further the investigations on the role of ROS in the relationship between viral infections with ADRD.

## 2. Reactive Oxygen Species

**Figure 3:**
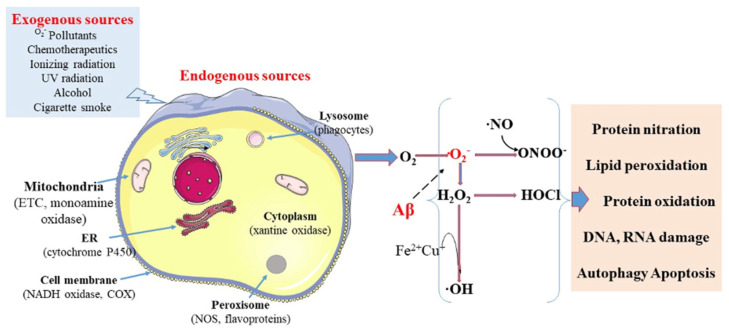
Schematics of the production and actions of ROS. Molecular oxygen (O_2_) is a free radical with two unpaired electrons but remains stable. One-electron reduction of O_2_ forms superoxide (•O_2_^−^), a radical species. Further reduction yields hydrogen peroxide (H_2_O_2_), a non-radical. Reduced metal ions, especially Fe^2+^, can donate another electron to H_2_O_2_ to produce a hydroxyl radical (•OH). Excessive production of ROS leads to oxidative stress, which contributes to the onset and progression of many human diseases, including AD. Both exogenous and endogenous sources generate ROS.

In this section, the principles of ROS biology are briefly introduced. The link between ROS and ADRS as well as viruses are described in Section 4 and Section 5, respectively. ROS are a broad class of highly reactive oxygen-containing molecules [16]. Oxidative stress is defined as an imbalance between the production of ROS and the body’s antioxidant defenses. This imbalance can result from excessive ROS production or inadequate antioxidant response [17,18,19]. ROS can damage proteins, lipids, DNA, and small molecules, resulting in a disruption of cellular function (Figure 3). Under normal physiological conditions, ROS serve as signaling molecules that regulate biological functions [20,21].

ROS can be classified as free radicals or non-radicals. Free radicals have one or more unpaired electrons, making them highly reactive. They are extremely unstable molecules that can react with a variety of molecules, including DNA, proteins, and lipids [22]. Free radicals include superoxide anion radicals (•O_2_**^−^**), hydroxyl radicals (•OH), nitric oxide (•NO), and nitrogen dioxide (•NO_2_) [22,23]. However, non-radicals are produced when two free radicals share their unpaired electrons [24,25,26]. Hydrogen peroxide (H_2_O_2_), hypochlorous acid (HOCl), ozone (O_3_), singlet oxygen (^1^O_2_), nitrous acid (HNO_2_), nitroxyl anion (NO^−^), hydroperoxides (ROOH), peroxynitrite (ONOO^−^), and aldehydes (HCOR) are among the non-free radical species [19,24].

The main source for its production is nonenzymatic electron transfer processes in which an electron is transferred to O_2_ [24]. Mitochondria are the organelles that generate the most intracellular ROS in the body, accounting for about 90% of all cellular ROS [27]. ATP production takes place in the process of oxidative phosphorylation, which is linked to the transfer of electrons through the electron transport chain (ETC) located in the inner membrane of mitochondria. In complex IV, electrons are transferred to O_2_, which undergoes a four-electron reduction, leading to the generation of water molecules. Single electrons may, however, leak during the processes involved in electron transport, especially from the ubiquinone and cytochrome c (i.e., electron carriers of the ETC). In complexes I or III of the ETC, the electrons react with O_2_ to form •O_2_**^−^**.

It has been estimated that 10% of ROS are made by enzymes involved in the functioning of the cell membrane and other organelles, besides mitochondria. These include monoamine oxidase in the mitochondria; NADPH oxidase and cyclooxygenases in the cell membrane; cytochrome P450 in the cell membrane, endoplasmic reticulum, and mitochondria; and xanthine oxidase in the cytoplasm [27,28,29].

Most potent ROS is the •OH, which is highly reactive with virtually any organic and inorganic molecules [24]. The •OH is primarily produced in the Fenton reaction, where H_2_O_2_ is reduced by metal ions like Fe^2+^. In the Haber-Weiss reaction, •O_2_**^−^** reduces Fe^3+^ to Fe^2+^, which then participates in the Fenton reaction to generate •OH from H_2_O_2_ [24,26].

## 3. Alzheimer’s Disease and Related Dementia (ADRD)

AD, frontotemporal dementia, Lewy body dementia, and vascular dementia are common neurodegenerative conditions that impair memory, cognition, and daily functioning, predominantly in the older population. AD is a progressive neurodegenerative disorder characterized by progressive cognitive decline, memory impairment, and loss of functional independence. Accounting for approximately 60–80% of dementia cases globally, AD poses a significant public health challenge. As global life expectancy increases, the prevalence of AD has risen substantially. Around 50 million people worldwide are currently living with dementia, a number expected to triple by 2050 [30]. Clinically, AD progresses through several stages, beginning with mild cognitive impairment and advancing to severe dementia (Table 1) [31]. In addition to cognitive decline, patients often experience impairments in performing basic activities of daily living, along with changes in mood, perception, and personality, collectively referred to as behavioral and psychological symptoms of dementia [32]. Understanding the underlying pathophysiology of AD is essential for the development of effective diagnostic tools and therapeutic strategies.

The hallmark neuropathological features of AD include extracellular amyloid-beta (Aβ) plaques, intracellular neurofibrillary tangles composed of hyperphosphorylated tau, neuroinflammation, and oxidative stress [33]. These abnormalities disrupt synaptic function, contributing to the progressive loss of cognitive function. Additional pathological mechanisms include acetylcholine deficiency, activation of the NLRP3 inflammasomes, and mitochondrial dysfunction [34].

Diagnosis of AD involves a combination of behavioral and cognitive assessments, neurological examinations, blood and cerebrospinal fluid analyses, evaluating biomarkers such as Aβ40/42 and p-tau181/Aβ42, and neuroimaging. Ongoing research aims to develop minimally invasive biomarkers and radiolabeled tracers for early detection, while also investigating Aβ aggregation, cerebral hypoperfusion, metabolic decline, and neuroinflammation [35].

While the exact etiology of AD remains unclear, multiple risk factors have been identified. AD is classified into familial and sporadic forms. Familial AD is generally caused by autosomal dominant mutations in the amyloid precursor protein, presenilin 1, and presenilin 2 genes. Sporadic AD, the more common form, has a multifactorial origin involving aging, genetic predispositions, environmental influences, and metabolic factors. The presence of the apolipoprotein E ε4 (APOE-ε4) allele is the strongest genetic risk factor for sporadic AD. Lifestyle choices and comorbid conditions such as cardiovascular disease, diabetes, and obesity also influence the risk of developing sporadic AD [36].

For many years, the amyloid hypothesis has dominated clinical trials and studies on AD [37]. Intracellular Aβ accumulation precedes extracellular plaque formation and involves toxic oligomer formation from initial monomers [38,39]. This intracellular Aβ accumulation results in synaptic abnormalities and cognitive decline [40,41].

Other pathophysiological theories for AD suggest that oxidative stress plays an important role in the disease course [17,42]. While ROS are essential in normal brain function, including synaptic plasticity and memory formation, an imbalance between ROS production, redox signaling, and antioxidant levels can result in neuronal damage and the advancement of disease [43]. According to proteomic research, cellular stress happens at the pre-plaque stage of AD-like amyloid pathology [44]. Oxidative damage to the brain causes changes in biomolecules (e.g., proteins, lipids, and nucleic acids) and tends to increase as people age.

Vascular dementia, the second most prevalent form of dementia, accounts for nearly 30% of cases. However, due to overlapping symptoms, it is often misdiagnosed as AD in elderly individuals. Vascular dementia is a progressive cognitive disorder resulting from chronic cerebrovascular dysfunction, leading to reduced cerebral blood flow and ischemia in the brain. This results in neuronal loss, white-matter damage, and cognitive decline. Common risk factors include vessel rupture, narrowing, and occlusion, which impair the brain’s ability to receive oxygen and nutrients.

Increasing evidence suggests that viral infections may contribute to the development and progression of AD and other forms of dementia [45]. Pathogens have been linked to the production and aggregation of Aβ peptides [14], supporting the infectious hypothesis, particularly for sporadic AD [46]. Neurotropic viruses can infect the central nervous system (CNS) and initiate or worsen neurodegenerative processes. Additionally, research shows that one of the key mechanisms linking viral infections to AD is oxidative stress mediated by ROS, which leads to neuronal damage. Viral infections can induce mitochondrial dysfunction and NADPH oxidase activation, producing excessive ROS. This oxidative stress may further enhance the amyloidogenic processing of amyloid precursor protein, promoting Aβ accumulation.

## 4. Roles of ROS in ADRD

Studies have shown that oxidative stress-induced damage is a major contributor to aging and, consequently, to the development of neurodegenerative diseases. Markers of oxidative stress, decline in antioxidant levels, and various mitochondrial DNA alterations have been observed in the brains of older people [47,48], individuals with mild cognitive impairment [49], Down syndrome [50,51], AD [17,52], and in transgenic animal models of AD [53,54]. Oxidative damage products, originating from lipids, DNA, and RNA, as well as glycation end products, have been detected in the blood, plasma, serum, cerebrospinal fluid, urine, and saliva of AD patients, where they are considered biomarkers of neurodegeneration and AD [55,56,57].

The brain, particularly neurons, is highly vulnerable to oxidative stress and the buildup of oxidative damage. This is mainly due to the brain’s high oxygen consumption, which leads to increased production of ROS, primarily at complex I of the ETC. Additionally, neuronal membranes are rich in polyunsaturated fatty acids, making them prone to lipid peroxidation. The brain’s high metabolic activity further contributes to elevated ROS production from mitochondria [58,59]. Brain tissue contains significant amounts of metals, especially iron, which promotes the generation of ROS through the Fenton reaction [60,61]. However, compared to other organs, the brain has a decreased ratio of antioxidant to prooxidant enzymes [60]. This imbalance increases oxidative damage. In the hippocampus tissues of post-mortem AD patients, our laboratory observed increased levels of carbonylated proteins [62].

Protein aggregation and its spread are key features of both familial and sporadic forms of AD. This aggregation process leads to excessive ROS production, resulting in oxidative stress and damage to vital macromolecules. ROS also contributes to mitochondrial dysfunction, neuroinflammation, and the breakdown of the blood–brain barrier, all of which accelerate AD progression [63]. Neuronal death can result from ROS-induced lipid peroxidation, which alters membrane fluidity, permeability, and transport functions. Studies have shown that intracellular Aβ generates ROS by interacting with cellular membranes, particularly through its methionine 35 residue, initiating lipid peroxidation [64]. Aβ also disrupts mitochondrial function by embedding itself in the mitochondrial membrane, lowering ATP production and membrane potential [65,66]. Furthermore, Aβ plaques inhibit cytochrome oxidase, disrupting the ETC and increasing oxidative stress [67,68]. ROS generated from this disrupted chain further promotes Aβ aggregation and tau pathology, reinforcing the cycle of ROS production. Additionally, Aβ binds to the receptor for advanced glycation end products, activating downstream signaling pathways that drive oxidative stress [69]. Aβ also interferes with NMDA receptor function, leading to calcium imbalance, neuronal hyperexcitability [70,71], and further oxidative stress [72].

AD and Parkinson’s diseases, as well as stroke, link to deficiencies in repairing chronic or acute oxidative DNA damage in neurons. While DNA damage accumulates naturally over time due to metabolism, acute stressors can cause rapid oxidative impairment. Due to their high metabolic rate, brain cells rely heavily on efficient DNA repair systems to maintain the integrity of DNA and dNTP pools [58,73]. Post-mitotic, terminally differentiated neurons lack robust replication-associated DNA damage detection and repair systems [74]. As a result, the brain’s genetic stability is heavily dependent on the base excision repair system. Research indicates that neurons rely on error-prone DNA repair processes like non-homologous end joining rather than replication-associated DNA repair [70,75]. When antioxidant enzymes involved in base excision repair are deficient, oxidative DNA damage accumulates, contributing to long-term neuronal dysfunction [73,76].

Oxidative damage not only contributes to the progression of AD but also appears early in the disease, before clinical symptoms emerge [75]. This supports the oxidative stress hypothesis of AD pathogenesis [17,77]. The oxidative stress hypothesis [78] has also been extended to other neurodegenerative diseases, including vascular dementia. ROS may play a more significant role in the beginning and progression of vascular dementia because oxidative damage happens early on or right after an ischemic episode [79].

## 5. Relationships Between Viral Infections and ADRD

In 1991, Jamieson and co-workers [80] identified herpes simplex virus 1 (HSV-1) DNA in the brains of AD patients. Over the past three decades, many viruses, such as various herpesviruses, HIV, flaviviruses, Borna virus, hepatitis viruses, influenza A virus, and SARS-CoV-2, have been connected to an elevated risk of AD. Nevertheless, only a small amount of research has been done on the possible involvement of many of these viruses in the pathophysiology of AD [81,82,83,84,85].

### 5.1. Herpes Simplex Viruses and ADRD

The potential connection between viruses and ADRD primarily stems from the idea that chronic infections can stimulate or worsen neuroinflammation and pathological changes in the brain. Among the most studied viruses in this context are herpesviruses, such as herpes simplex virus type 1 (HSV-1) and human herpesvirus 6 (HHV-6). Research shows that members of the Herpesviridae family, including human cytomegalovirus (CMV), HSV-1, varicella-zoster virus (VZV), HHV-6, human herpesvirus 7 (HHV-7), Kaposi’s sarcoma-associated herpesvirus, and Epstein–Barr virus (EBV), may contribute to AD by disrupting cellular homeostasis and dysfunction, and with microglia via virus–host protein–protein interactions. These interactions impact key pathological processes such as ROS, Aβ formation, neuronal death, gliogenesis, and autophagy, all of which are commonly linked to neurodegenerative diseases [86].

Viral reactivation, such as that of latent HSV-1, has been associated with increased Aβ production. According to the antimicrobial protection hypothesis, Aβ may function as an innate immune defense, capable of binding and neutralizing microbial pathogens, including viruses [87]. Supporting this theory, Aβ oligomers have been shown to trap herpesviruses within amyloid plaques, potentially preventing their spread in neural tissue. For example, Eimer et al. [88] demonstrated that Aβ rapidly fibrillates around HSV-1 particles both in vitro and in vivo, effectively entrapping the virus within amyloid plaques in the brains of infected transgenic mice. Similarly, post-mortem analyses of human AD brains have revealed colocalization of HSV-1 DNA with amyloid plaques, suggesting that reactivation of latent herpesvirus infection may contribute to amyloid deposition [89,90]. This hypothesis is further supported by data from Readhead et al. [91], who identified elevated levels of human herpesvirus 6A (HHV-6A) and HHV-7 in AD brains, accompanied by altered expression of amyloid precursor protein processing genes. Together, these findings support the concept that Aβ may act as an antimicrobial peptide that, through aggregation, entraps herpesviruses and other pathogens. While this protective mechanism could serve to limit infection, it may also promote the accumulation of amyloid plaques, thereby linking microbial infection to the pathogenesis of AD [87]. Thus, the defensive mechanism becomes maladaptive over time, contributing to plaque accumulation and neurotoxicity.

**Figure 4:**
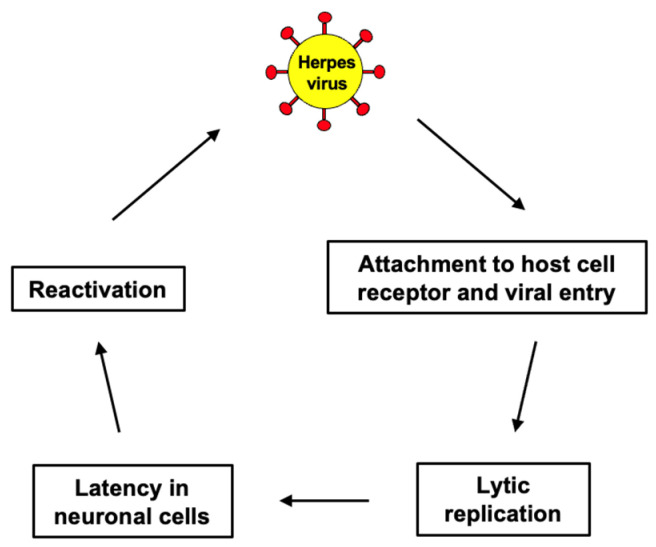
HSV-1 infects cells by attaching to cellular receptors and entering the host cell. The virus then undergoes a lytic replication or establishes latency in neuronal cells. The latent virus can reactivate and re-enter the lytic phase.

The mechanisms by which viruses interact with neural tissues can provide insight into their potential contributions to neurodegenerative pathology. Initially, viruses such as HSV-1 attach to specific receptors on neuronal membranes, facilitating entry into cells. For HSV-1, entry is mediated by binding to heparan sulfate proteoglycans and subsequent fusion with the neuronal membrane. This targeted invasion allows viruses to infiltrate brain regions critical to memory and cognition, such as the hippocampus. After initial infection, neurotropic viruses can establish latency within neurons, evading immune responses (Figure 4). Reactivation of latent viruses, potentially triggered by stress or immunosuppression, can lead to repeated cycles of viral replication and inflammation. Chronic inflammation is a well-established factor in the progression of AD, and reactivated viral infections may exacerbate this process. Viral infections activate microglia and astrocytes, leading to the release of pro-inflammatory cytokines. Persistent neuroinflammation has been implicated in the accumulation of Aβ plaques and tau protein tangles, hallmark features of AD pathology, as well as in neuronal and synaptic dysfunction, contributing to cognitive decline in conditions like frontotemporal dementia and dementia with Lewy bodies. Viral replication and protein synthesis, as well as oxidative stress induced by viral activity, may damage neurons, contributing to neuronal loss and cognitive decline.

The viral hypothesis of AD proposes that HSV-1 may play a causative role in the development of the disease, particularly in individuals carrying the APOE-ε4 allele, with estimates suggesting it could contribute to up to 60% of sporadic AD cases [90]. Epidemiological data indicate that by the age of 70, over 80% of the population is latently infected with HSV-1 [89,92]. Wozniak et al. [89] provided more direct evidence of an association between HSV-1 and AD pathology by demonstrating that HSV-1 DNA is specifically localized within Aβ plaques in the brains of AD patients, but not in control brains, using highly sensitive in situ PCR techniques. The authors reported that up to 90% of Aβ plaques examined in AD brains contained HSV-1 DNA. However, many contradictory data can be found in the published literature in this regard. Many studies show that infection rates do not differ significantly between AD patients and age-matched controls, nor between APOE-ε4 carriers and non-carriers, suggesting that infection alone is insufficient to drive disease onset [89,93]. HSV-1 DNA has been detected in the brains of both AD patients and controls at similar frequencies, typically in latent form within trigeminal ganglia and brain tissue [80]. Intriguingly, some studies observed that among controls, HSV-1 was more frequently detected in non-APOE-ε4 carriers, although small sample sizes limit the strength of this conclusion [93]. Furthermore, studies employing immunohistochemistry for HSV-1 proteins in brain tissue reported negative results, as the viral protein load in latently infected neurons is expected to be orders of magnitude lower than in acutely infected cell cultures, making detection challenging [94].

Beyond detection, mechanistic studies have provided important insights. HSV-1 infection has been shown to impair lysosomal function, a key process implicated in AD pathogenesis. Lysosomes are essential for the degradation of misfolded proteins, including Aβ, and their dysfunction contributes to Aβ accumulation and neuronal damage [95]. In vitro studies have demonstrated that HSV-1 infection increases lysosomal load and induces lysosomal membrane permeabilization, especially under conditions of oxidative stress, thereby promoting neurodegenerative cascades [96]. Additionally, HSV-1 has been reported to enhance glycogen synthase kinase-3β (GSK-3β) activity, facilitating tau hyperphosphorylation, another hallmark of AD [96]. Together, these findings support a model in which HSV-1 acts not merely as a passive passenger in the aging brain but as an active participant in neurodegeneration, particularly in genetically susceptible individuals.

Recent studies have demonstrated that, in contrast to HSV-1, VZV is not directly implicated in AD pathology. In an in vitro model, HSV-1 quiescence was induced in human induced neural stem cells through a two-step protocol: cells were infected with HSV-1 for 24 h, followed by treatment with 100 μM valacyclovir for an additional 24 h. This antiviral intervention suppressed productive infection, as evidenced by the absence of infectious progeny production, and induced expression of the latency-associated transcript, confirming the establishment of a latent-like state. Valacyclovir effectively prevented active HSV-1 replication and inhibited Aβ accumulation in the cultures. However, subsequent challenge of these quiescent cultures with VZV resulted in HSV-1 reactivation, marked by a significant increase in HSV-1 gene expression, enhanced Aβ deposition, and morphological evidence of neuronal injury, including neurite retraction and cell body shrinkage, as verified by immunocytochemical and immunofluorescent staining [97].

While AD remains the most prevalent form of dementia, other neurodegenerative dementias, including vascular dementia, frontotemporal dementia, and dementia with Lewy bodies, are also characterized by pathogenic protein misfolding. In frontotemporal dementia and tauopathies, the TAR DNA-binding protein 43 inclusions underlie neuronal degeneration, while dementia with Lewy bodies is distinguished by the accumulation of misfolded alpha-synuclein in Lewy bodies [98]. Recent studies have expanded the role of herpesviruses beyond AD, highlighting their possible contribution to these other dementias. For instance, HSV-1 DNA has been detected in post-mortem brain tissue of vascular dementia patients at significantly higher rates than in controls, with viral load correlating with microvascular damage and white matter hyperintensities [99]. Similarly, HHV-6 infection has been shown to potentiate tau hyperphosphorylation through upregulation of glycogen synthase kinase-3β activity in neuronal cultures, offering a plausible mechanism for herpesvirus-induced tauopathy relevant to frontotemporal dementia [100]. In the context of dementia with Lewy bodies, experimental models have demonstrated that HSV-1 infection can accelerate alpha-synuclein aggregation and enhance its prion-like spread along neural circuits, potentially facilitating the progression of synucleinopathies. Moreover, high-throughput sequencing studies have identified distinct herpes viral genomic signatures and virus-induced immune transcriptomic changes in brain samples from individuals with various non-AD dementias, suggesting virus-driven neuroinflammatory processes may represent a common pathological thread [91,101]. Collectively, these findings imply that herpesviruses may not only trigger but also exacerbate protein misfolding and neurovascular injury, contributing to the complex multifactorial etiology of multiple dementia subtypes.

Both HSV-1 and herpes simplex virus type 2 (HSV-2) infections are linked to an increased risk of dementia, including AD and vascular dementia. A nationwide Taiwanese cohort study found that HSV-infected patients had about a 2.5-fold higher risk of developing dementia compared to uninfected controls. HSV-1 showed a particularly strong association with AD, while HSV-2 was more linked to vascular dementia. Importantly, treatment with anti-herpetic medications, such as valacyclovir or acyclovir, was associated with a markedly reduced risk of dementia, suggesting that viral reactivation and related neuroinflammation may contribute to neurodegeneration, that antiviral therapy could be protective [102].

### 5.2. Varicella-Zoster Virus (VZV) and ADRD

VZV (Human herpesvirus 3), also known as Herpes zoster virus, is one of the most common viruses encountered by humans and causes two distinct diseases: chickenpox (varicella) and shingles (herpes zoster). The clinical manifestation of the initial infection, which primarily affects young children, especially those of preschool age, is chickenpox. After a primary infection of chickenpox, VZV establishes latency in the dorsal root ganglia, where it remains dormant within nerve cells. Reactivation later in life leads to herpes zoster, characterized by the development of acute neuritis and post-herpetic neuralgia. Acute neuritis occurs in many individuals with localized zoster, except in young children. Post-herpetic neuralgia can develop in up to 50% of adults, depending on age. Treating both acute neuritis and post-herpetic neuralgia can be challenging for individual patients.

A meta-analysis including nine studies with a total of 3,326,673 participants found that VZV infection was associated with an increased risk of developing dementia [103]. In studies rated as moderate quality, the pooled data showed an even higher risk. Several potential mechanisms have been proposed to explain how a neurotropic virus, zoster, might contribute to dementia, including neuroinflammation, injury to cerebral blood vessels, and direct neuronal damage. However, some epidemiological studies have not strongly supported these links. At the end of one study’s follow-up period, dementia was diagnosed in 9.7% of zoster patients and 10.3% of matched controls. Subgroup analysis revealed no significant increase in the long-term risk of dementia, except possibly in individuals with CNS infections. When analyzing AD as a separate outcome, similar results were observed.

The association between VZV reactivation and subsequent risk of dementia is pronounced in cases where zoster involves the cranial nerves, particularly ophthalmic zoster, or the CNS. Given the neurotropic nature of VZV and its known predilection for cranial nerve ganglia and CNS tissues, these subtypes may carry a greater risk of neuroinflammatory or neurodegenerative sequelae. Notably, Schmidt et al. [104] found that individuals with cranial nerve or CNS involvement showed stronger associations with AD compared to those with zoster in other anatomical regions [104]. This suggests a potential pathway whereby localized viral reactivation within or near the brain may exacerbate neuronal damage, inflammation, or protein misfolding processes implicated in AD pathology. These findings demonstrate the importance of recognizing ophthalmic or neuroinvasive zoster as clinically relevant subtypes that may warrant closer neurological monitoring or even antiviral interventions to mitigate long-term cognitive risks.

Gene expression analysis of VZV-infected human brain vascular adventitial fibroblasts revealed significant enrichment of pathways involved in vascular remodeling, including those associated with vascular diseases. RNA sequencing showed notable upregulation of gene sets implicated in amyloidosis, diabetes mellitus, and AD. Analytical modeling identified several key upstream regulators, including amyloid precursor protein, apolipoprotein E (APOE), microtubule-associated protein tau, presenilin 1, and islet amyloid polypeptide (amylin) [105]. At three days post-infection, immunofluorescent staining confirmed the presence of VZV glycoprotein, indicating active viral infection, as well as amyloidogenic peptides Aβ42 and amylin, detected using specific antibodies. Additionally, intracellular amyloid aggregates were visualized using Thioflavin T staining, which binds to β-sheet-rich fibrillar structures. Mock-infected cells showed none of these markers, confirming that the findings were infection-driven. Follow-up studies showed increased secretion of matrix metalloproteinases-3 and -10, along with enhanced migration of both infected and uninfected cells treated with conditioned media from VZV-infected cultures. Further pathway analysis revealed enrichment in signaling networks associated with amyloid-related diseases, tauopathies, and other progressive neurological disorders. The intracellular buildup of Aβ, amylin, and amyloid fibrils suggests that these fibroblasts may contribute to the weakening of cerebral vessel walls, heightened inflammation, and possibly the development of vascular dementia or AD-like pathology [105].

Complementing these lab findings, a large Swedish registry-based cohort study (2005–2017) examined whether symptomatic HSV or VZV infection was linked to dementia, and whether antiviral treatment was protective. This epidemiological study followed over 265,000 individuals aged 50+, comparing those with herpes diagnoses (and/or antiviral prescriptions) to matched controls. The key findings included that herpes infection without antiviral treatment was linked to an increased risk of dementia, while antiviral treatment, such as with valacyclovir, was associated with a reduced risk. Among those diagnosed with herpes, treated individuals had a 25% lower risk of dementia compared to untreated peers. Though the study supports a potential protective role for antivirals, the authors caution that, as an observational study, it cannot prove causality [106].

### 5.3. Cytomegalovirus (CMV) and ADRD

CMV (Human herpesvirus 5) may contribute to the pathogenesis of AD through chronic immune activation and viral reactivation in aging individuals [14]. CMV, a persistent beta-herpesvirus that establishes lifelong latency, can undergo periodic reactivation, particularly in immunosenescent hosts. Only about 10% of primary CMV infections are symptomatic, typically in older children and adults, while the remaining 90% remain asymptomatic. Viral reactivations have been associated with a marked expansion of CD8^+^ T lymphocytes displaying a CD28^−^/CD57^+^ phenotype, a recognized marker of terminal differentiation and cellular senescence [107]. This subset of senescent T cells has been implicated in age-related immune dysfunction and is increasingly observed in both CMV-seropositive individuals and AD patients, suggesting a potential mechanistic link between CMV-induced immunosenescence and neurodegeneration [108].

Emerging evidence indicates a similar expansion of CD4^+^CD28^−^/CD57^+^ T cells in CMV-infected individuals, which may also contribute to Aβ accumulation [109,110]. Although these senescent CD4^+^ T cells are less proliferative, they retain cytotoxic capabilities and secrete high levels of pro-inflammatory cytokines. This creates a persistent, low-grade inflammatory environment, a known risk factor for AD. Among these inflammatory mediators, interferon-gamma (IFN-γ) has drawn particular attention as a central pro-inflammatory cytokine involved in AD immunopathology. IFN-γ enhances microglial activation and upregulates major histocompatibility complex expression in the CNS, while also promoting the release of neurotoxic mediators and potentially exacerbating tau phosphorylation and Aβ pathology. Collectively, these findings support a multifaceted model in which CMV-driven immune aging and chronic pro-inflammatory T cell responses may accelerate or intensify the neuropathological processes underlying AD, especially in genetically susceptible or immunologically compromised individuals.

A growing body of clinical evidence demonstrates that viral infections influence AD and related dementias through measurable biomarker changes in CSF, plasma, and other biofluids (Table 2).

### 5.4. SARS-CoV-2 and ADRD

Given its global impact, SARS-CoV-2 has recently been a major focus of research on virus-induced neurodegeneration. This section summarizes emerging evidence indicating that SARS-CoV-2 may contribute to ADRD [111,112]. While this issue is highly important clinically, available evidence does not yet establish a causal link between SARS-CoV-2 infection and ADRD.

Coronaviruses are positive-sense single-stranded RNA viruses that are generally responsible for causing the common cold [113,114]. However, some strains can be lethal, as seen in the COVID-19 pandemic, which was caused by SARS-CoV-2 [115,116]. Globally, hundreds of millions have been infected, with over seven million deaths reported, leading to significant health, economic, and sociological consequences. SARS-CoV-2 uses angiotensin-converting enzyme 2 (ACE2) as a receptor to enter the host cells [117,118]. ACE2 is an enzyme that degrades angiotensin II, a major mediator in cardiovascular as well as neurological pathology, and its interaction with the virus is linked to complications in COVID-19 patients [116,119,120]. These interactions may exacerbate vascular dysfunction and promote neuroinflammatory cascades, processes central to both COVID-19 and AD progression [112].

**Figure 5:**
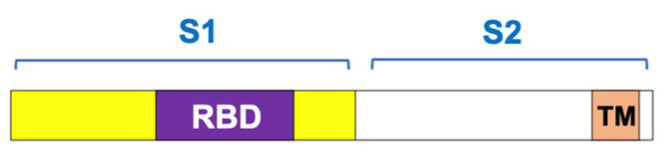
Structure of SARS-CoV-2 spike protein. The spike protein consists of S1 and S2 subunits. The S1 subunit contains the receptor binding domain (RBD) that binds to the host cell receptor, and the S2 subunit contains the transmembrane (TM) domain that anchors the protein to the viral membrane.

**Figure 6:**
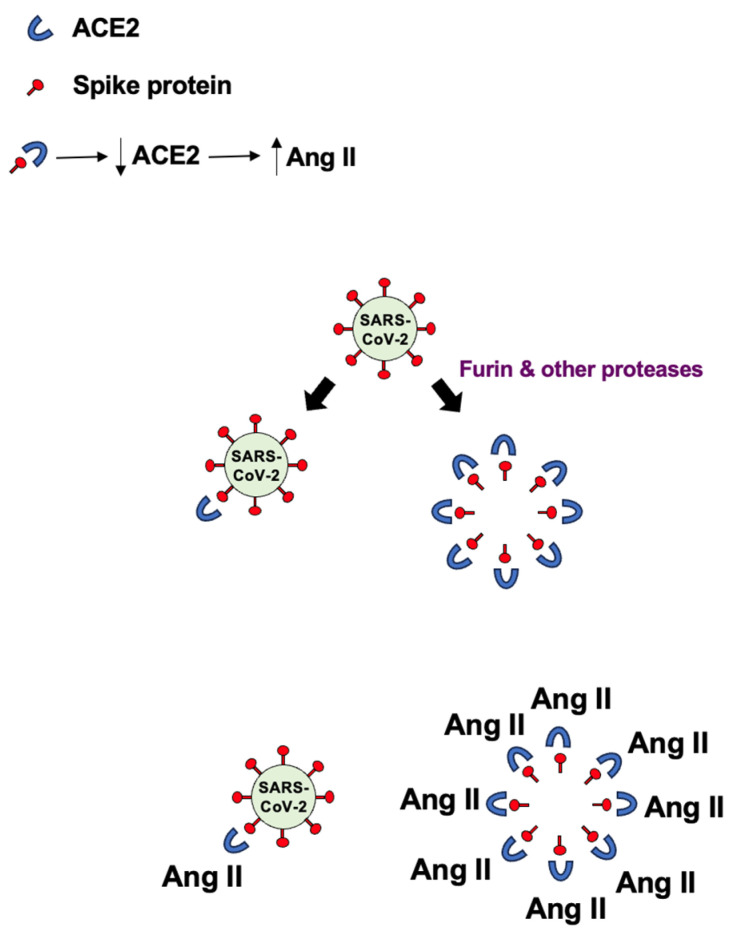
Amplification of biological effects due to the production of freely circulating spike proteins. We propose that viral fusion proteins detaching from the virus and circulating freely in the blood amplify the biological responses, exacerbating complications of viral infections. This scheme illustrates how the SARS-CoV-2 spike protein may enhance the upregulation of angiotensin II (Ang II). A coronavirus particle contains approximately 50–100 spike protein trimers. Adapted from Ayyubova et al. [9].

The virus’s spike protein, which facilitates cell entry, consists of two subunits: S1, which contains the receptor-binding domain that attaches to ACE2, and S2, which anchors the protein to the viral membrane and helps fuse with host cell membranes [117] (Figure 5). The process of SARS-CoV-2 entering human host cells is regulated by the cleavage between S1 and S2, which is carried out by proteases like furin and transmembrane protease, serine 2 [121]. This cleavage can also release the S1 subunit into the blood, potentially leading to medical complications [8,122]. The amount of circulating spike protein also increases after COVID-19 vaccination, such as when the body produces spike protein through mRNA in the recipient’s cells [123,124]. We proposed that the production of free S1 spike protein could exacerbate effects on ACE2 and other targets, since each SARS-CoV-2 virus particle carries 50 to 100 spike protein trimers on its surface [9], as depicted in Figure 6.

Lung cells are the primary targets of SARS-CoV-2, resulting in severe pneumonia and acute respiratory distress syndrome [125,126]. It has been noted that certain populations of infected individuals are more vulnerable to being severely affected by COVID-19. Elderly patients are particularly susceptible to developing severe and fatal conditions [116,119,127], suggesting that this virus also affects organs other than the respiratory system.

While much of the focus has been on treating the lung and cardiovascular aspects of COVID-19 to reduce mortality, it has been found that COVID-19 patients also develop many neurological manifestations [128,129]. Mao et al. [128] conducted a retrospective study of 214 hospitalized patients with laboratory-confirmed COVID-19 in Wuhan, China, and reported that about 36.4% of hospitalized COVID-19 patients exhibited neurological symptoms. These included CNS issues such as headaches, dizziness, impaired consciousness, ataxia, seizures, cognitive dysfunctions, as well as encephalitis, acute cerebrovascular disease, ischemic stroke, etc. Peripheral nervous system involvement was also common, including cranial polyneuritis, Guillain–Barré syndrome, nerve pain, neuro-ophthalmological and neuromuscular junction disorders, neurosensory hearing loss, and skeletal muscle injury.

Further supporting this link, a UK Biobank imaging study of 785 participants (ages 51–81) showed that even mild COVID-19 can cause noticeable grey matter loss in areas like the orbitofrontal cortex and parahippocampal gyrus, both critical for olfactory and memory functions. The study also found widespread tissue damage in areas functionally connected to the primary olfactory cortex, as well as increased overall brain atrophy and measurable cognitive decline. These changes persisted after controlling for variables like hospitalization, emphasizing that even mild infections can lead to detectable and potentially lasting neuroanatomical changes. These alterations mirror the brain regions affected in early AD and support the hypothesis that SARS-CoV-2 may initiate or accelerate neurodegenerative processes through mechanisms such as olfactory deprivation, inflammatory responses, and disruption of vascular and glial support systems. This imaging-based evidence reinforces the growing concern that COVID-19 may not only worsen preexisting neurological vulnerabilities but could also serve as a catalyst for the onset of AD-like pathology, particularly in older or at-risk individuals [130]. However, the study, like many observational investigations it references, does not fully disentangle the role of confounding factors. Comorbidities such as hypertension, diabetes, and obesity are established risk factors for both dementia and COVID-19 severity, and socioeconomic status can shape access to healthcare, infection risk, and baseline cognitive reserve. Without careful adjustment for these variables, it remains difficult to determine whether the observed brain changes reflect direct consequences of SARS-CoV-2 infection or whether they are amplified by preexisting dementia risk factors.

Patients with more severe COVID-19 infections are significantly more likely to experience neurological complications. The association between COVID-19 severity and neurological involvement is supported by the pro-inflammatory and neurotoxic effects of cytokine storms that characterize severe cases [111]. Impacts on the CNS may trigger or exacerbate the underlying neurodegenerative conditions, including AD [112]. Supporting these clinical observations, a study by Frontera et al. [131] compared serum neurodegenerative biomarkers in hospitalized COVID-19 patients without prior cognitive impairment to non-COVID controls with normal cognition, mild cognitive impairment, or AD. This study found that hospitalized COVID-19 patients exhibited significantly elevated levels of neurodegenerative biomarkers, including total tau, phosphorylated tau-181 (p-tau181), glial fibrillary acidic protein, and neurofilament light chain, especially among those with encephalopathy or who succumbed to the illness. Remarkably, these biomarker levels exceeded those found in non-COVID individuals with established AD, indicating that SARS-CoV-2 infection can induce AD-like neurodegenerative changes [112]. Furthermore, these elevations strongly correlated with disease severity and clinical outcomes, underlining the virus’s potential to cause or accelerate neuronal and glial injury [131].

SARS-CoV-2 infects brain astrocytes in COVID-19 patients. Crunfli et al. [132] found that infection of these cells can impair neuronal variability, indicating that the virus not only infects the brain but also disrupts essential cellular processes required for neuronal health, leading to cognitive impairments. These disturbances are mechanistically supported by the potential for virus-induced neuroinflammation to aggravate pre-existing neurodegenerative pathologies, including AD [111]. This is especially concerning for individuals already at risk for cognitive decline, as neuroinflammation and vascular damage can exacerbate pre-existing neurodegenerative conditions [112].

It has also been reported that dementia increases the risk of severe COVID-19 threefold [133,134,135,136], and the mortality rate is 30% higher in individuals with dementia [134,135]. Specifically, Bianchetti et al. [137] observed that dementia patients with COVID-19 often present with atypical symptoms and delayed recognition of the virus, which contributes to delayed treatment and worsened outcomes. The increased risk of poor outcomes in dementia patients is partly due to the overlap between neurological dysfunction and other health challenges, such as weakened immune responses and underlying cardiovascular conditions [133]. Impaired immune responses, comorbidities, and existing CNS vulnerabilities make individuals with dementia particularly susceptible to both severe infection and poor recovery [111].

A large-scale study by Hampshire et al. [138] assessed over 113,000 UK participants and revealed that COVID-19 infection, especially in individuals with long-lasting symptoms, was associated with persistent deficits in memory, reasoning, and executive function. Many participants reported experiencing “brain fog,” and objective testing showed cognitive performance declines like early-stage AD. To reinforce this behavioral evidence, Radke et al. [139] conducted proteomic and transcriptomic profiling of brainstem, cerebellum, and olfactory tissues from early- and late-phase COVID-19 cases. Their findings revealed immune activation (type I interferon signaling), microglial and astrocytic alterations, and downregulation of synaptic and myelination pathways, even in the absence of active viral replication. These molecular disturbances affect key brain regions involved in cognition and olfaction, offering mechanistic explanations for COVID-related cognitive decline.

Evidence continues to accumulate that SARS-CoV-2 not only worsens preexisting neurodegeneration but may also initiate it. Epidemiological studies have suggested that SARS-CoV-2 infection increases the risk of developing AD [140,141]. Several studies have proposed a mechanistic link between AD and COVID-19 [142,143,144]. Reiken et al. [142] demonstrated that brains of COVID-19 patients exhibit pathological signaling patterns like those observed in AD, including leaky ryanodine receptor 2 calcium channels, tau hyperphosphorylation, and increased amyloid precursor protein processing. These molecular disruptions promote synaptic dysfunction, oxidative stress, and neuronal damage. Their findings suggest that SARS-CoV-2 can trigger AD-like signaling cascades in the brain, even in patients without prior neurological disease, raising concerns about long-term neurodegenerative consequences of the infection. COVID-19 may also trigger or accelerate amyloid-β deposition and promote cognitive impairment through neuroinflammatory and oxidative mechanisms [111]. Blood–brain barrier breakdown associated with COVID-19 may further exacerbate neuronal injury and permit entry of peripheral immune cells and inflammatory factors into the brain [111]. The APOE e4 genotype, a known genetic risk factor for AD, has been linked to more severe COVID-19 outcomes [145]. Individuals with this genotype are particularly vulnerable to both severe COVID-19 and accelerated AD pathology, highlighting the importance of monitoring these individuals closely.

Duff et al. [15] further indicate this crucial connection. They found that individuals with confirmed COVID-19 infection exhibited changes in plasma biomarkers associated with AD, including a reduced Aβ42:Aβ40 ratio and elevated pTau-181 levels. These biomarker changes correlated with brain imaging findings and cognitive decline, suggesting that even mild-to-moderate COVID-19 may contribute to long-term dementia risk. Jiang et al. [146] observed similar electrophysiological abnormalities in both COVID-19 survivors and AD patients, including altered brainwave patterns and disrupted neural synchrony, further supporting the idea of SARS-CoV-2-associated neurodegenerative disease. A study by Shan et al. [147] revealed that individuals aged 60 and older who contracted COVID-19 have an elevated risk of developing new-onset dementia, with this risk persisting for up to 24 months post-infection. This prolonged vulnerability highlights that COVID-19-related cognitive decline may not be transient but could initiate the development of progressive neurodegeneration in the aging population. Additionally, these findings, combined with reports of shared electrophysiological abnormalities between COVID-19 survivors and AD patients, suggest that SARS-CoV-2 infection may act as a catalyst for cognitive decline in individuals at risk for dementia [111,147].

Xu et al. [148] performed a major cohort study involving over 154,000 COVID-19 survivors compared to 11 million controls. It was revealed that SARS-CoV-2 infection was associated with a nearly twofold increased risk of new onset AD and a 77% increase in memory problems within a year of infection. These elevated risks were also present among non-hospitalized individuals, indicating that even mild COVID-19 cases can have long-term neurological consequences. Overall, this study concluded that approximately 7% of COVID-19 survivors experience lasting neurological complications, symptoms closely resembling those seen in early AD, and mild cognitive impairments.

### 5.5. Zika Virus and ADRD

Zika virus, a mosquito-borne member of the Flavivirus genus, was declared an international public health emergency by the World Health Organization in 2016 [149]. Its genome is a positive-sense single-stranded RNA, approximately 10 kilobases long, enclosed within an icosahedral capsid. The genome encodes three main structural proteins: capsid (C), pre-membrane/membrane (prM/M), and envelope (E). It is flanked by two untranslated regions: the 5′-UTR and 3′-UTR. It also encodes several non-structural proteins involved in viral replication, including NS1, NS2A, NS2B, NS3, NS4A, NS4B, and NS5 [149]. The Zika virus has been associated with neurological disorders such as Guillain–Barré syndrome and microcephaly. It may also interact with the cellular amyloid precursor protein, inhibiting the beta-site amyloid precursor protein cleaving enzyme 1 binding site, which is essential for amyloid precursor protein cleavage. This disruption could lead to amyloid precursor protein accumulation. Interestingly, amyloid precursor protein appears to function as a negative regulator of Zika virus replication in both human neural progenitor/stem cells and neonatal mouse brain cells, suggesting a potential protective mechanism that limits virus-induced brain damage. Costa et al. [150] also pointed out the relationship between the Zika virus and AD, demonstrating that memantine, a drug that inhibits NMDA receptors and is FDA-approved to treat AD, may reduce neurodegeneration caused by the Zika virus. More recently, Zika virus infection has been shown to accelerate AD-like phenotypes in brain organoids derived from human-induced pluripotent stem cells [151].

### 5.6. Enterovirus and ADRD

The genus Enterovirus includes a group of small, positive-sense, single-stranded RNA viruses, divided into ten species of true enteroviruses and three species of rhinoviruses. These viruses are responsible for a wide range of illnesses, from mild conditions like the common cold to more severe diseases such as poliomyelitis and aseptic meningitis [152,153]. Enterovirus 71 has emerged as a significant public health concern, particularly in Asia, due to its association with hand, foot, and mouth disease and its potential to cause severe neurological complications. The brainstem is the primary site of infection, and in young children, Enterovirus 71 infection can result in serious clinical outcomes and long-term psychological disorders [154]. Some studies suggest a potential link between enterovirus infections and neurodegeneration, including AD, through the activation of toll-like receptors (TLRs). TLRs initiate inflammatory responses that are implicated in various pathological conditions, including neurodegeneration. Additionally, the unfolded protein response and oxidative stress, both associated with TLRs, are features commonly observed in neurodegenerative diseases. It has been proposed that Enterovirus 71 infection may trigger pathogenesis in the brain via the TLR7 pathway, contributing to neurodegeneration in both human and animal models.

Interestingly, recent findings have revealed that Aβ functions as an antimicrobial peptide. Aβ deposition may initially serve as part of the innate immune response to infection. While early Aβ production might help defend against pathogens, chronic or reactivated infections could lead to harmful accumulation over time. Research has demonstrated that Aβ1-42 significantly inhibits Enterovirus 71 during the early stages of its life cycle, such as viral attachment and uncoating, in various cell lines (e.g., neural cells). Furthermore, Enterovirus 71 infection also stimulates the production and accumulation of Aβ in SH-SY5Y cells, suggesting a complex interplay between the virus and Aβ that may influence the development or progression of neurodegenerative diseases [155]. Enterovirus D68 infection has been linked to several neurological complications, including acute flaccid myelitis, cranial nerve dysfunction, encephalitis, and meningoencephalitis. These complications typically occur following initial symptoms such as fever, respiratory issues (e.g., coughing, rhinorrhea, and pharyngitis), and gastrointestinal symptoms (e.g., vomiting and diarrhea). In some Enterovirus D68 cases, cranial nerve dysfunction has been observed without limb paralysis. The cranial nerve motor nuclei control muscles in areas such as the face, oral cavity, respiratory tract, and tongue. Dysfunction in these areas can result in various motor-related neurological symptoms. It is possible that Enterovirus D68 infects cranial neurons directly or enters the CNS through mechanisms like those used in infecting anterior horn motor neurons in the spinal cord, due to its tropism for motor neurons. Additionally, the detection of Enterovirus D68 in cerebrospinal fluid suggests that the virus may cross the blood–brain barrier to access the CNS, facilitating its spread and potentially contributing to neurological complications. While it remains unclear whether crossing the blood–brain barrier directly results in spinal cord gray matter infection, another possibility is that Enterovirus D68 infects lymphocytes or other immune cells, which then migrate into the CNS and spread the virus [156]. Recent findings also indicate that Enterovirus 71 infection leads to mitochondrial dysfunction, including alterations in mitochondrial morphology and distribution within cells. These dysfunctional mitochondria become significant sources of ROS generation, contributing to cellular damage and neurodegeneration. These changes are linked to disruptions in cellular energy metabolism. Mitochondrial ROS also plays a crucial role in viral replication, and the observed increase in mitochondrial mass in infected cells may be a compensatory response to the energy deficit caused by mitochondrial damage [157].

### 5.7. HIV and ADRD

**Figure 7:**
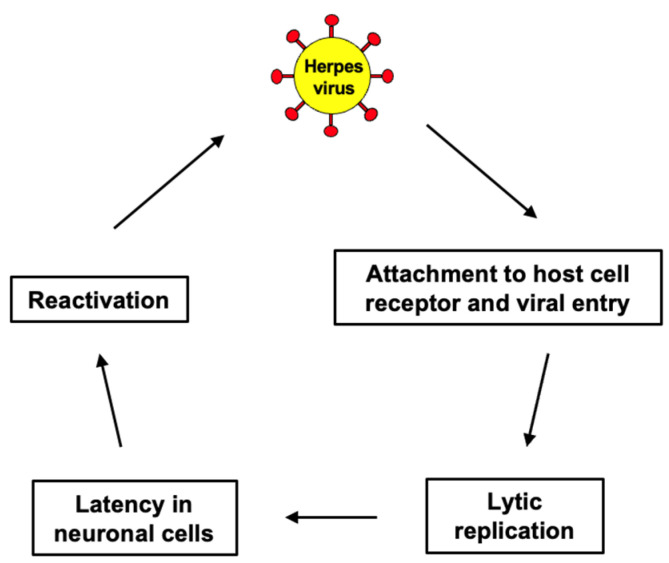
HIV life cycle. The steps in the HIV life cycle include host cell binding, membrane fusion, reverse transcription, integration into the host genome, gene transcription and replication, viral assembly, and budding to exit the host cell.

HIV is a retrovirus whose viral fusion protein gp160 binds to the human CD4 receptor to facilitate fusion and introduce viral RNA into the host cells. Viral reverse transcriptase converts RNA into DNA, and the integrase incorporates the HIV genome into host DNA (Figure 7). CD4, the primary receptor for HIV-1, is mainly expressed on the surface of immune cells, as well as on vascular endothelial and smooth muscle cells [158,159]. Gp160 also binds to co-receptors CCR5 and CXCR4 [160]. It can be cleaved into gp120, which contains receptor-binding sites, and gp41, which anchors to the viral membrane, resulting in circulating gp120 in the bloodstream [161,162]. Circulating gp120 levels of 1–8 nM have been detected in patients with HIV [163,164], including those receiving antiretroviral therapy with undetectable viral loads. Gp120 has been shown to activate cell signaling pathways in various cell types [159,165,166,167,168].

Antiretroviral therapies have significantly extended life expectancy for people living with HIV [169,170]. As a result, there has been an increase in non-communicable diseases within this population [169,171,172]. HIV-associated neurocognitive disorders affect 30% to 60% of individuals with HIV [173,174]. The worldwide prevalence of asymptomatic neurocognitive impairment, mild neurocognitive disorder, and HIV-associated dementia has been reported as 23.5%, 13.3%, and 5.0%, respectively, in HIV-infected adult populations [175]. While HIV-related complications are multifactorial, gp120, shed from the virus, contributes to neuronal and endothelial damage [176,177]. Damage to these cells can lead to stroke and subsequent vascular dementia [178,179,180]. Stroke is a leading cause of disability and death worldwide [181], with 25–30% of stroke survivors developing dementia and an even larger proportion experiencing cognitive impairment [182]. The incidence of stroke is similar between males and females [183]. Approximately 87% of all strokes are ischemic, caused by reduced blood flow leading to brain cell death and neurological deficits. HIV, along with hypertension [181], is a known risk factor for stroke. The incidence of stroke is higher in people with HIV than in uninfected individuals [184,185,186,187,188]. Vascular risk factors play a significant role in the development of HIV-associated dementia and cognitive decline [189].

Additionally, a 24-week pilot trial of lamivudine (3TC), an HIV nucleoside reverse transcriptase inhibitor, in patients with mild cognitive impairment due to suspected AD showed the drug to be feasible, safe, and cerebrospinal fluid-penetrant. While cognition remained stable, exploratory biomarkers revealed favorable trends, including reduced glial fibrillary acidic protein levels measured in cerebrospinal fluid (neuroinflammation marker) and increased plasma A β42/40 ratio (suggestive of reduced amyloid burden). These findings support the rationale for repurposing nucleoside reverse transcriptase inhibitors as potential disease-modifying therapies in ADRD and highlight the need for larger randomized controlled trials to confirm efficacy [190].

## 6. Cellular Production of ROS by Viruses

In this section, we will review experimental evidence for the cellular production of ROS in response to viruses. Two mechanisms will be addressed: (i) the production of ROS triggered by viral infection and (ii) ROS generation induced by free viral membrane fusion proteins.

### 6.1. Cellular Production of ROS by HSV-1 and HSV-2

The ability of HSV-1 infection to produce oxidative stress was first reported in 1994 by Palù et al. [191] who detected lipid peroxidation in HeLa S3 cells infected with HSV-1 by measuring the levels of malondialdehyde via reaction with thiobarbituric acid. The authors demonstrated a time-dependent increase in malondialdehyde following HSV-1 infection. To our knowledge, the first direct observations of ROS production in HSV-1 infection were published in 2007 and 2008. Kavouras et al. [192] showed that HSV-1 infection of neuronal-like P19 embryonal carcinoma cells (differentiated with retinoic acid) increased intracellular ROS and released lipid peroxidation by-products into the culture medium. After inoculation with the HSV-1 strain KOS, they measured ROS at 1, 2, 3, and 24 h post-infection using hydroxyphenyl fluorescein, which fluoresces upon oxidation by •OH. ROS levels were significantly elevated by 21.5%, 69.8%, 45.7%, and 80%, respectively, compared to controls. Heat- or UV-inactivated HSV-1 did not produce ROS. HSV-1 replication was inhibited by ebselen (a glutathione peroxidase mimetic), suggesting that H_2_O_2_ is required for HSV-1 replication. Similarly, Aubert et al. [193] showed that HSV-1 infection of Jurkat T cells caused a marked increase in ROS detection by 2′,7′-dichlorofluorescein diacetate. By contrast, infection with the glycoprotein J deletion virus caused only a little increase in ROS, indicating the role of glycoprotein J in HSV-1-mediated ROS production. ICP27 is an immediate early protein of HSV-1. Kim et al. [194] reported that ICP27-expressing Vero monkey kidney epithelial cells exhibited increased ROS production compared to control Vero cells devoid of ICP27. HSV-1 infection of Vero cells has also been shown to produce ROS [195,196].

In mouse models of HSV-1 encephalitis, oxidative stress markers were detected [197], and vitamin E deficiency worsened disease pathology [198]. ROS production and oxidative damage induced by HSV-1 in cultured microglial cells from wild-type C57B/6 mice were reduced in cells from TLR-2 knockout mice [199]. As early as 2005, peptides from HSV-2 glycoprotein G were shown to promote •O_2_^−^ production via phagocyte NADPH oxidase in human neutrophils and monocytes [200,201]. In 2011, the functional role of HSV-derived ROS was confirmed by Hu et al. [202], who reported that HSV-1-induced cytokine and chemokine production in murine microglial cells was reduced by NADPH oxidase inhibitors. Similarly, antioxidants *N*-acetylcysteine and pyrrolidine dithiocarbamate inhibited HSV-2-induced cytokine expression in murine macrophages [203]. Marino-Merlo et al. [204] reported that HSV-1 infection produced ROS in wild-type U937 monocytic cells but not in U937 cells expressing a dominant-negative IκB-mutant, suggesting the role of NF-κB in HSV-1-mediated ROS production. Natural antioxidants such as Mori ramulus from the mulberry branches used in traditional Chinese medicine [195], embelin from berries of the Embelia ribes plant found in India [196], Korean chestnut honey [205], and polyphenols from the heartwood of Maackia amurensis, widespread in the far east of Russia [206] have been shown to protect against HSV-1 by their antioxidant activities.

Additionally, HSV-1 has been increasingly linked to promoting ferroptosis, a nonapoptotic, iron-dependent form of programmed cell death characterized by the accumulation of lipid peroxides and ROS. Recent findings by Xu et al. [207] provide compelling evidence that HSV-1 induces ferroptosis in both human astrocytes and microglia. In a mouse model, pharmacological inhibition of ferroptosis significantly reduced HSV-1 encephalitis, indicating a functional role of this cell death pathway in viral neuropathogenesis. Mechanistically, HSV-1 disrupts the cellular redox balance by enhancing the activity of Kelch-like ECH-associated protein 1 (KEAP1), an E3 ubiquitin ligase adaptor that promotes the degradation of nuclear factor erythroid-2–related factor 2 (Nrf2), the master transcription factor of the antioxidant defense system. By suppressing Nrf2 activity, HSV-1 downregulates key target genes such as SLC7A11 involved in cystine uptake for glutathione synthesis, and GPX4, a lipid peroxide-detoxifying enzyme essential for ferroptosis resistance. This disruption impairs glutathione metabolism, increases intracellular iron and lipid ROS accumulation, and primes cells for ferroptotic death. Furthermore, neuronal cultures infected with HSV-1 show increased lipid peroxidation and greater vulnerability to ferroptosis inducers like erastin. These observations highlight HSV-1’s capacity to generate cytotoxic ROS and promote ferroptosis susceptibility, particularly in neurons, under oxidative stress or co-pathological conditions.

In addition to the studies of ROS in human herpes virus as described above, there have been several published studies on non-human herpesvirus, in particular, bovine herpesvirus 1 (BHV-1). BHV-1 belongs to the family Herpesviridae and the subfamily Aphaherpesvirinae and infects cattle of all ages and breeds. BHV-1 infection of cattle results in clinical problems in the upper respiratory tract, nasal cavity, ocular cavity, and reproductive system [208,209]. Researchers at Yangzhou University in China found that BHV-1 infection increased ROS levels in MDBK bovine kidney epithelial cells, measured using 2′-7′-dichlorodihydrofluorescein diacetate. Both *N*-acetylcysteine and the NADPH oxidase inhibitor diphenylene iodonium reduced viral replication [208].

In a subsequent report, these investigators described that BHV-1 infection decreased the levels of glutathione, and the exogenous administration of glutathione inhibited BHV-1 replication [210]. In these cells, BHV-1 infection also activated cell signaling pathways, including phospholipase C, which was found to be involved in the mechanism of ROS production. BHV-1 also activated mitogen-activated protein kinases (MAPK) such as p38MAPK, JNK, and Erk1/2, which also participate in viral replication. However, *N*-acetylcysteine did not block the activation of these kinases, suggesting that ROS are not involved in these pathways [211]. In Zhu et al. [212], the authors described that BHV-1 infection promotes oxidative DNA damage as detected by the production of 8-hydroxyguanine in MDBK cells, as well as in A549 human lung epithelial cells and U-2 OS human bone epithelial cells. In MDBK cells, BHV-1 infection decreased mRNA expression of SOD1, catalase, and glutathione peroxidase 4, while SOD2 mRNA level was increased [209]. BHV-1 infection also decreased protein expression levels of redox regulators such as Nrf2, heme oxygenase-1, and NADPH quinone oxidoreductase-1 [213]. A research group in Brazil demonstrated that BHV-1 infection triggers ROS production in Neuro2a mouse neuroblastoma cells and C6 rat glial cells [214]. In live animals, Tafuri et al. [215] reported that Mediterranean buffaloes (Bubalus bubalis) seropositive to Bubaline herpesvirus 1 (closely related to BHV-1) exhibited higher oxidative stress than seronegative animals. Equine herpesvirus type 1, another alpha herpesvirus that causes respiratory disease in horses, was shown to induce ROS in E. Derm horse skin fibroblasts [216] and primary murine neurons [217], as monitored with the CellROX.

### 6.2. Cellular Production of ROS by VZV

Early in vitro studies demonstrated that treatment of polymorphonuclear leukocytes with VZV significantly increased the production of H_2_O_2_, indicating direct ROS production by the virus [218,219]. Further clinical validation was provided by Khazan et al. [220] in a case–control study involving 43 patients with herpes zoster and 47 age-matched healthy controls. The study revealed a significant elevation in both total oxidant status and oxidative stress index in herpes zoster patients, alongside a marked reduction in total antioxidant capacity and total polyphenol content, compared to controls. These findings provided evidence that VZV infection disrupts redox homeostasis, favoring a pro-oxidative cellular environment characterized by excessive ROS accumulation.

### 6.3. Cellular Production of ROS by CMV

Ikeda et al. [221] reported that intranasal infection of immunocompetent mice with murine CMV increased xanthine oxidase activity. Administration of the xanthine oxidase inhibitor allopurinol and a •O_2_^−^ scavenger SOD reduced CMV-induced pulmonary lesions. The authors concluded that •O_2_^−^ plays a role in CMV-associated pneumonitis. Speir et al. [222] reported that infection of human coronary artery smooth muscle cells with human CMV led to intracellular ROS generation and activation of the NF-κB transcription factor. Since *N*-acetylcysteine inhibited CMV-induced NF-κB activation, the authors proposed that ROS contributes to CMV’s effects on host cells. They also later reported that estrogen has antioxidant properties that counteract CMV’s impact on human coronary artery smooth muscle cells [223].

Kim et al. [224] identified insoluble substances associated with human CMV-infected cells that induced death in certain immune cells, but not in fibroblasts or astrocytoma. These substances triggered ROS production, which was inhibited by NADPH oxidase inhibitors. Combs et al. [225] found that human foreskin fibroblasts infected with human CMV released ROS by upregulating mitochondrial ETC proteins essential for viral replication. Similarly, Wang et al. [226] reported ROS production in WI-38 human fetal lung fibroblasts following human CMV infection.

### 6.4. Cellular Production of ROS by Coronaviruses

The ability of coronaviruses to promote ROS production has been well characterized since the outbreak of SARS-CoV-1 in 2003, and, more recently, SARS-CoV-2 in 2019. Coronaviruses have been shown to activate ROS-producing enzymes, such as NADPH oxidases and xanthine oxidases, while concomitantly weakening antioxidant defense systems [227].

Several studies have shown that nonstructural and accessory proteins of SARS-CoV-1 can trigger intracellular ROS accumulation. Li et al. [228] demonstrated that SARS-CoV-1 papain-like protease induces intracellular ROS production in human lung epithelial A549 cells, as detected by flow cytometry with the use of 2′,7′-dichlorofluorescein diacetate. ROS generation triggered activation of the p38 MAPK and STAT3 pathways, leading to upregulation of the transcription factor Egr-1 and increased expression of transforming growth factor β and other pro-fibrotic genes. These effects were attenuated by ROS inhibitor YCG063, confirming a ROS-dependent mechanism. Similarly, Lin et al. [229] reported that expression of SARS-CoV-1 3C-like protease in human promonocyte HL-CZ cells led to a significant increase in intracellular ROS, as detected by dihydrorhodamine 123 fluorescence, alongside caspase-3/9-dependent apoptosis and NF-κB activation. Chen et al. [230] reported that open reading frame 8a, an accessory protein encoded by SARS-CoV-1 because of a 29-nucleotide deletion in the viral genome, localizes to the mitochondria in hepatocellular epithelial HuH-7 cells, where its overexpression elevated ROS levels, as measured by 2′,7′-dichlorofluorescein diacetate. This overexpression also caused hyperpolarization of mitochondrial membrane potential and activated caspase-3-mediated apoptosis. In monkey kidney fibroblast COS-1 cells, Zhang et al. [231] found that the nucleocapsid protein induced apoptosis via the mitochondrial pathway under serum starvation. Flow cytometric analysis using 2′,7′-dichlorofluorescein diacetate revealed increased intracellular ROS levels, accompanied by loss of mitochondrial membrane potential, release of cytochrome c, and activation of caspases 3 and 9. These effects were absent in HepG2 or Huh-7 cells, and neither the matrix nor spike proteins induced apoptosis in any of the three cell lines. Altogether, these studies demonstrate that SARS-CoV-1 induces oxidative stress through a variety of viral components, including both structural and nonstructural components.

**Figure 8:**
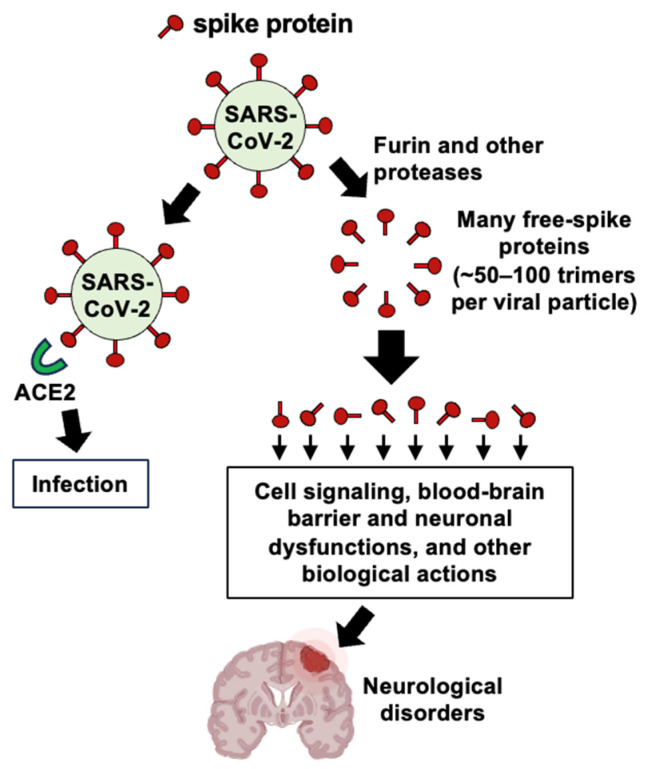
The amplification of biological effects due to the production of freely circulating SARS-CoV-2 spike proteins. During COVID-19 virus infection, one set of spike protein trimers of the SARS-CoV-2 virus interacts with one ACE2 molecule. Circulating SARS-CoV-2 particles, each containing ~50–100 trimers of spike proteins, could release many free spike protein fragments, such as S1 proteins through the actions of extracellularly located proteases. Evidence suggests that proteases, such as furin, are detected in the plasma of COVID-19-positive individuals. These free spike proteins can activate cell signaling and contribute to blood–brain barrier and neuronal dysfunctions, and other pathological effects, potentially leading to neurological disorders. Adapted from Ayyubova et al. [9].

In the blood circulation of individuals positive for COVID-19, many free S1 spike proteins can be produced by extracellularly occurring proteases such as furin [232,233,234], since each SARS-CoV-2 viral particle carries 50–100 spike proteins [9]. This amplification mechanism may have harmful effects on the brain (Figure 8). Multiple studies have also shown that the SARS-CoV-2 spike protein can cause oxidative stress, cellular senescence, and inflammation in various human cell types. Clough et al. [235] treated human microglial HMC3 cells with recombinant SARS-CoV-2 spike protein and observed increased intracellular ROS production, measured using the oxidative stress indicator CM-H2DCFDA. The authors found that spike protein treatment increased mitochondrial fragmentation, quantified with MitoTracker Red, and boosted apoptotic signaling, detected using a caspase-3/7 green reagent. In ARPE-19 retinal pigment epithelial cells, SARS-CoV-2 spike protein induced ROS and cellular senescence through p53/p21 signaling, as shown by Zhang et al. [236]. Antioxidant treatment with *N*-acetylcysteine reversed these effects, supporting the role of ROS in cellular senescence that may contribute to age-related macular degeneration, a condition linked to dementia. Sun et al. [237] demonstrated that spike protein made male Sprague–Dawley rats more sensitive to angiotensin II-induced hypertension by increasing neuroinflammation and oxidative stress in the hypothalamus.

In human umbilical endothelial cells, Zhang et al. [238] discovered that the SARS-CoV-2 spike protein increased ROS production through NOX2/NOX4 activation, with a synergistic rise under hyperglycemic conditions. The results suggest spike-induced oxidative stress as a possible mechanism for vascular complications in diabetic COVID-19 patients. Meyer et al. [239] examined how spike protein exposure affected human endothelial (TMNK-1, EAhy926) and epithelial (A548, Huh7.5) cells using a ROS detection kit. The spike protein raised ROS levels in both cell types and activated senescence markers (p16 and p21). It also caused a senescence-associated secretory phenotype, releasing proinflammatory molecules like interleukin 6 and HMGB1. Conditioned media from spike-expressing epithelial cells also induced paracrine senescence in endothelial cells, increasing leukocyte adhesion by upregulating vascular cell adhesion molecule 1 and intracellular adhesion molecule 1. These effects were reduced by inhibitors blocking interleukin 6 signaling (e.g., tocilizumab) and inflammatory pathways (e.g., zanubrutinib). Wang et al. [240] found that SARS-CoV-2 spike protein increased ROS levels in thrombin-activated human platelets, leading to mitochondrial depolarization and lipid peroxidation. This oxidative stress caused platelet hyperactivity and aggregation. Coenzyme Q10 significantly lowered ROS levels and restored antioxidant defenses, pointing to a potential therapeutic role in COVID-19-related thrombotic complications.

In A549 human alveolar basal epithelial cells, Lesiów et al. [241] demonstrated that SARS-CoV-2 spike protein synergizes with copper ions to generate mitochondrial ROS, resulting in single- and double-strand DNA breaks. Oxidative stress induced by the spike protein and copper ion was confirmed by immunofluorescent staining, suggesting that trace metal interactions may worsen lung injury. Greenberger et al. [242] demonstrated that exposure to the SARS-CoV-2 spike protein induces oxidative stress and DNA damage in both human lung epithelial cells as well as K18-hACE2 transgenic mouse lung cells. Specifically, they observed increased ROS, DNA double-stranded breaks, and activation of transforming growth factor β signaling pathways. These molecular changes led to cellular senescence and fibrotic remodeling, resembling the effects of ionizing radiation. Additionally, immune-mediated inflammatory amplification further contributed to ROS production. Treatment with the antioxidant MMS350 significantly reduced oxidative damage, highlighting a potential therapeutic strategy for preventing COVID-19-associated pulmonary fibrosis.

In cardiomyocytes (AC16 cells), Huynh et al. [243] showed that short-term spike protein exposure increased mitochondrial respiration and ATP production, while prolonged exposure (about 72 h) led to mitochondrial dysfunction, calcium overload, mitochondrial fragmentation, and elevated mitochondrial ROS levels. These effects were ACE2-dependent and suggest a mechanism for cardiac injury in COVID-19 patients. A similar study by Van Tin et al. [244] showed how SARS-CoV-2 spike protein enhanced ROS production and fibrotic marker expression in human cardiac fibroblasts via ACE2-NF-κB signaling. Increased oxidative stress of cardiac fibroblasts was confirmed by fluorescence-based ROS assays, thereby linking spike-induced ROS to cardiac fibrosis. Finally, Bojkova et al. [245] confirmed that SARS-CoV-2 infection triggers oxidative stress responses and apoptosis in human induced pluripotent stem cell-derived cardiomyocytes. RNA sequencing analysis demonstrated upregulation of pathways related to oxidative stress and apoptotic signaling, which aligns with cardiac injury commonly observed in patients.

Expanding on this, Ahn et al. [246] demonstrated that the spike protein activates NADPH oxidase 2 (NOX2) in both human and murine macrophages via protein kinase C signaling, resulting in increased ROS levels. The authors employed a dichlorofluorescein assay to determine intracellular ROS production, flow cytometry to assess mean fluorescence intensity, and scanning electron microscopy to quantify membrane ruffles. The observed spike protein-NOX2 interaction may contribute to macropinocytosis-mediated viral entry by bypassing ACE2, suggesting that oxidative stress supports alternative viral uptake routes. Furthermore, neutrophils can also be contributors to spike protein-mediated oxidative stress. Almeida et al. [247] studied that both the Wuhan and Omicron spike variants induced ROS production in human neutrophils, with the Omicron variant stimulating a stronger response. The formation of immune complexes between the Wuhan spike protein and IgG1 antibodies further enhanced ROS and myeloperoxidase release, whereas similar complexes with the Omicron variant had a reduced effect. These findings were able to indicate that variant-specific immune complex formation can influence oxidative responses as well as contribute to the varied inflammatory profiles seen within a range of SARS-CoV-2 variants.

Spike protein also promotes cellular senescence through ROS-driven DNA damage. Almeida et al. [247] showed that immune complexes formed between the spike protein and antibodies, such as IgG1, enhanced ROS generation in neutrophils. This response differed by variant, with the Omicron spike protein causing stronger ROS activity on its own, while the Wuhan spike protein effects were increased in the presence of antibodies. In addition to direct and immune-mediated ROS production, spike protein expression also initiates oxidative stress in surrounding cells via paracrine signaling. Meyer et al. [237] showed that conditioned media from spike protein-expressing epithelial cells can trigger ROS generation and senescence in neighboring endothelial cells.

**Figure 9:**
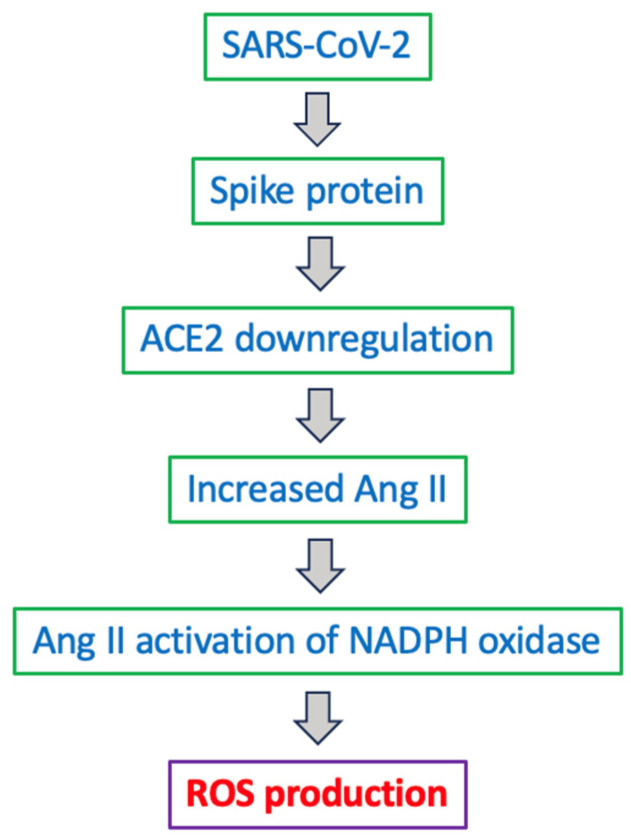
One possible mechanism of how SARS-CoV-2 may produce intracellular ROS. The spike protein (either bound to the viral particle or free) promotes the internalization and the reduction in plasma membrane ACE2, resulting in increased angiotensin II (Ang II) in the blood circulation. Ang II is a well-known ligand that activates NADPH oxidase-mediated ROS production.

The SARS-CoV-2 spike protein causes ACE2 downregulation [248,249,250,251,252], resulting in uncontrolled accumulation of angiotensin II [253,254,255], a vasoactive peptide that promotes vasoconstriction, inflammation, and tissue fibrosis [256,257,258]. Angiotensin II also stimulates the intracellular production of ROS [259] that promotes redox signaling and oxidative stress, which are key factors in disease development, progression, and the aging process [260]. One might postulate a mechanism of the SARS-CoV-2 spike protein as depicted in Figure 9, in which ACE2 downregulation-mediated increase in angiotensin II activates NADPH oxidase and ROS production.

While many studies use recombinant spike protein to isolate its effects, others employ live SARS-CoV-2 or pseudovirus infection models. In HEK293T, HEK293T-hACE2, Vero E6, 16HBE, and HMEC-1 cells, Li et al. [261] showed that spike pseudovirions upregulated intracellular ROS and suppressed the PI3K/AKT/mTOR signaling pathway. This suppression led to enhanced autophagy, apoptosis, and increased production of inflammatory cytokines. According to Xie et al. [262], live SARS-CoV-2 infection has been able to stimulate multiple ROS-dependent cell death pathways, such as apoptosis, NETosis, necroptosis, ferroptosis, pyroptosis, and autophagy in many host cells. These results were shown in both in vitro models, like infected Vero E6 cells, and in vivo systems, including hamster lung and human heart tissues. SARS-CoV-2 can also stimulate excessive ROS production through mechanisms such as mitochondrial dysfunction, NADPH oxidase activation, and inhibition of antioxidant responses, establishing ROS as a central mediator of cytotoxicity in viral replication and immune activation. Kelesidis et al. [263] contributed that the ApoA-I mimetic peptide 4F significantly reduced mtROS levels in SARS-CoV-2-infected Calu-3 and Vero E6 cells. This intervention strategy also inhibited viral entry and replication, exemplifying that targeting ROS may serve to limit both viral spread and oxidative damage.

Yu et al. [264] experimented that during infection, the SARS-CoV-2 nucleocapsid protein directly enhances mitochondrial ROS production. This occurs through mitochondrial localization of the nucleocapsid protein, stabilization of mitochondrial transcription factors, and upregulation of complexes I and III of the ETC, leading to impaired ATP synthesis and oxidative stress. The nucleocapsid protein also disrupts antioxidant defense mechanisms, particularly by inhibiting catalase activity via direct interaction and reducing SOD and glutathione peroxidase activity. Chen et al. [265] showed that viroporin 3a activates the NLRP3 inflammasome in lipopolysaccharide-primed murine macrophages through a mechanism requiring mitochondrial ROS, potassium efflux, and inflammasome assembly. This highlights the role of SARS-CoV-2-related viral proteins in initiating oxidative and inflammatory responses in macrophages.

Additional studies support ROS involvement in viral entry and pathogenesis. Chaubey et al. [266] highlighted that excess iron worsens SARS-CoV-2 severity by increasing ROS production in infected cells. Similarly, Mo et al. [267] found that SOD protects plasma cells from SARS-CoV-2-induced plasma cell apoptosis via ROS modulation. The authors performed an ELISA-based detection of ROS levels and flow cytometry for intracellular ROS measurement.

### 6.5. Cellular Production of ROS by Zika Virus

In mice, Zika virus infection increased testicular ROS levels. Treatment with ebselen, a glutathione peroxidase mimetic, improved testicular pathology and prevented the sexual transmission of the virus [268]. Zika virus infection of cultured U87-MG human glioblastoma cells or HepG2 hepatocellular carcinoma cells also increased ROS, lipid peroxidation, and protein carbonylation [269]. More recently, Wang et al. [270] demonstrated robust ROS production in Zika virus-infected RD human rhabdomyosarcoma cells and A549 human lung epithelial cells, implicating both mitochondrial dysfunction and NADPH oxidase activation as sources of ROS.

### 6.6. Cellular Production of ROS by Enteroviruses

Enteroviruses like poliovirus and coxsackieviruses have been shown to induce ROS. In SK-N-SH human neuroblastoma cells, Tung et al. [271] found that the enterovirus 71 infection triggered ROS generation. The use of NADPH oxidase inhibitors (apocynin and diphenyleneiodonium chloride), *N*-acetylcycteine, and p47phox siRNA effectively reduced the viral load. Cheng et al. [157] showed that enterovirus 71 infection caused mitochondrial ROS production in SF268 human glioblastoma cells. In African green monkey kidney epithelial Vero cells, enterovirus 71 infection led to ROS generation as detected by dichlorofluorescein fluorescence [272] and Cell ROX Deep Red [273]. Cheng et al. [273] further reported that ROS production involves interactions between the enteroviral 2B protein, a type II viroporin, and host cell mitochondrial voltage-dependent anion channel 3 (VDAC3). Cui et al. [274] found that enterovirus 71 infection induced the production of ROS in primary astrocytes as well as in U251 human glioblastoma, SK-N-MC human neuroblastoma, and A549 human epithelial cell lines, as monitored by the dichlorofluorescein fluorescence. In human neuroblastoma SK-N-SH cells infected with enterovirus 71, mitochondrial ROS was detected by MitoSOX [275]. Lipid peroxidation was induced by enterovirus 71 infection in human rhabdomyosarcoma cells [276]. ROS produced in response to infection of cultured cells with enterovirus 71 have been shown to participate in the mechanism of apoptosis [277,278,279].

In mice, coxsackievirus B3 infection increased the •O_2_^−^ production [280] and upregulated NOX4 expression in cardiomyocytes affected by viral myocarditis [281]. ROS generation was also observed in H9c2 rat heart myoblasts exposed to the virus [282,283], suggesting a potential role in the pathogenesis of myocarditis. Burg et al. [284] found that the loss of NADPH oxidase-derived •O_2_^−^ protected mice from coxsackievirus B-induced diabetes. In HeLa cells, coxsackievirus B3 promoted mitochondria ROS production as determined by MitoSOX [285]. Recently, Li et al. [286] linked coxsackievirus B3-induced viral myocarditis to cardiomyocyte ferroptosis, a ROS-dependent cell death mechanism.

### 6.7. Cellular Production of ROS by HIV

ROS play a role in HIV pathogenesis, particularly by acting as second messengers in NF-κB-mediated gene transcription [287]. As HIV was discovered, early evidence suggested the occurrence of oxidative stress in HIV-1-infected patients, such as increased production of malondialdehyde [288] and decreased thiol levels [289]. Among various HIV components, the role of gp120 has been implicated by several mechanistic studies. Gp120 is a glycoprotein coat found on the surface of HIV that plays a great role in neuronal cognitive impairment via ROS generation, release of cytokines, NMDA receptor overactivation, increased intracellular calcium levels, and reduced levels of nerve growth factor and glucose utilization, leading to neurotoxicity and neuronal death [290]. Additionally, gp120 is also linked to thrombotic and vascular diseases because it stimulates human arterial smooth muscles, leading to the coagulation cascade through the expression of tissue factor that is triggered and activated by ROS production [159].

In 1996, Shatrov et al. [291] reported that gp120 induces intracellular formation of H_2_O_2_ in Jurkat T cells. Foga et al. [292] also showed that gp120 increased thiobarbituric acid-reactive substances (TBARS) in U937 human myeloid leukemia cells. Antioxidants, including catalase, SOD, α-tocopherol, and deferoxamine, inhibited this increase. In rat Kupffer cells treated with gp120, •O_2_^−^ was found to be produced by measuring the reduction of cytochrome c [293]. Viviani et al. [294] observed that gp120 exposure led to 30% neuronal cell death in primary rat glial cultures, alongside H_2_O_2_ production (detected by measuring dichlorofluorescein fluorescence), which was blocked by the antioxidant Trolox. These results suggest the role of ROS in HIV exacerbation. Brooke et al. [295] reported increased glutathione peroxidase activity in cortical neurons exposed to gp120, indicating ROS involvement in HIV neurotoxicity.

Moreover, exposure of the primary human neuronal tissue cultures to gp120 was associated with astrocyte hypertrophy and hyperplasia, which causes dementia due to the upregulation of oxidative stress markers like inducible nitric oxide synthase (iNOS). Nitric oxide generated by gp120 can react with •O_2_^−^ to form ONOO^−^, a known neurotoxic oxidant. Additionally, nitric oxide can compete with cytochrome oxidase to interfere with mitochondrial cellular respiration, leading to oxygen depletion and the release of apoptotic markers [296]. Jana et al. [297] reported that the oxidative effect of gp120 incubated with primary neurons causes neuronal apoptosis by the production of ceramide, which results from the degradation of sphingomyelin by neutral sphingomyelinase enzyme. Also, gp120 induces NADPH oxidase-mediated production of •O_2_^−^, leading to oxidative stress. Interestingly, gp120 and Tat have been associated with alteration of the integrity of the blood–brain barrier to increase its permeability to drugs and toxins. Gp120 and Tat in HIV induce oxidative stress in the endothelial cell line from rat brain capillaries (RBE4) to decrease glutathione (GSH), oxidized glutathione (GSSG), catalase, and glutathione reductase antioxidant enzymatic activity that is reversed by *N*-acetylcysteine amide, which has a strong antioxidant protective effect against gp120 in RBE4 [298]. Gp120 was associated with Mn-SOD overexpression in astrocytes, a known antioxidant enzyme that neutralizes •O_2_^−^, suggesting the strong oxidative stress effect of gp120 on astrocytes, more than the neurons [299]. Yao et al. [300] stated that rat primary neurons exposed to both gp120 and cocaine cause an increase in proapoptotic markers, such as caspase-3 activity and Bax, and alteration of mitochondrial membrane potential that is reversed by NADPH oxidase inhibitors, such as apocynin.

The research on the production of ROS in HIV has become a very broad field, and recent review articles describe this specific topic [301,302]. Since the present article seeks to comprehensively cover the link between ROS and various viruses, and the space is limited, we refer to review articles as well as many others published over the years for specific issues related to HIV.

## 7. Conclusions, Limitations, and Future Directions

There is growing evidence suggesting that ROS play a critical role in linking viral infections to the development and progression of AD and other forms of dementia. A recent report suggesting that even mild-to-moderate COVID-19 may contribute to AD-related diseases [15] warrants serious attention from both the scientific community and the public. Many neurotropic and systemic viruses, such as HSV-1, CMV, Zika virus, enteroviruses, HIV, and SARS-CoV-2, trigger ROS production in infected cells. This occurs through mechanisms such as mitochondrial dysfunction, activation of NADPH oxidase, and suppression of host antioxidant defenses. The resulting oxidative stress promotes a cascade of damaging effects, including neuroinflammation, protein misfolding, synaptic loss, and neuronal death, all key features of neurodegenerative diseases like AD. HSV-1 can establish long-term latency in neurons, creating a persistent source of oxidative stress that may accelerate AD and other dementia subtypes.

Further, the connection between viral infections and neurodegenerative diseases offers promising avenues for research and therapy. Antiviral treatments, vaccines, and other interventions aimed at reducing neurological alterations could provide novel strategies for preventing or mitigating dementia progression across its various forms. Additionally, longitudinal studies are necessary to elucidate the causal relationships between viral infections and specific forms of dementia. By understanding these connections, researchers may uncover shared pathological pathways and develop more effective, broadly applicable therapeutic approaches.

Current evidence does not support establishing a causal relationship between viral infections and AD. Most existing data come from human observational studies, which we have incorporated into our review. To our knowledge, there are currently no clinical trials specifically aimed at suppressing or preventing the progression of neurodegenerative diseases through the modulation of viral infection–associated pathways. While this area remains largely unexplored in the clinical setting, both human observational studies and animal model experiments, such as those described in this review, provide compelling evidence that viral infections, including SARS-CoV-2, may accelerate or exacerbate AD pathology. These findings highlight the urgent need to promote and expand research efforts in this direction, with future studies bridging preclinical animal models and well-designed human trials to evaluate whether targeting viral mechanisms can alter the course of neurodegenerative diseases.

The influence of sex and age on ROS responses and viral susceptibility in neurodegeneration remains an important but underexplored aspect. In the reviewed studies, clear distributions by sex and age were not consistently reported, and in several cases, only male animals were studied, which limits the current evidence. This lack of inclusion hinders the ability to generalize findings, especially given known sex differences in AD prevalence and progression, as well as potential sex-specific immune responses to viral infection. Similarly, aging is a crucial factor in ROS biology and susceptibility to viral infections like COVID-19, yet few studies stratify outcomes by age in a way that provides mechanistic insights. These gaps apply to both the COVID-19-related data and the ROS-focused studies summarized in this review. We acknowledge that sex- and age-dependent effects are not sufficiently addressed in the literature and identify this as a limitation that requires focused investigation in future research.

**Figure 10:**
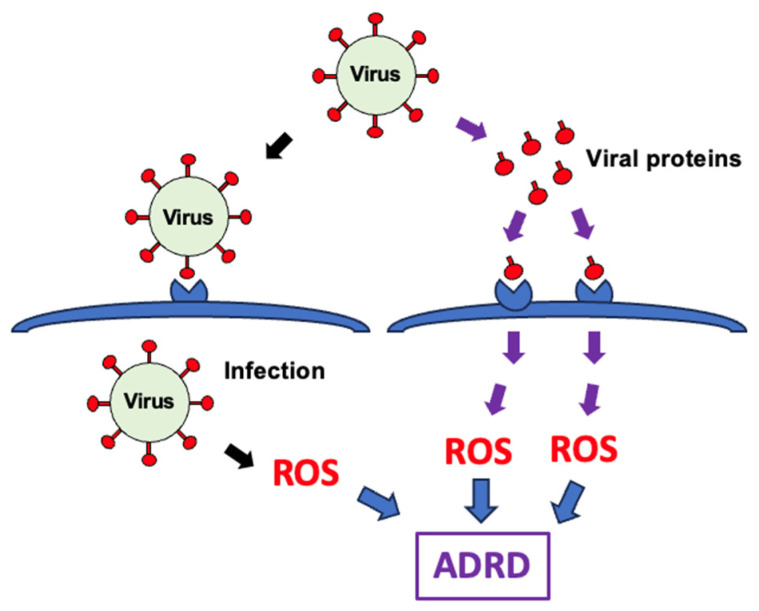
Scheme depicting viral infection and virus-associated complications with the proposed role of ROS in virus-associated pathogenesis of AD and other forms of dementia. Virus-mediated complications can arise from the direct effects of viral infection, including the death of host cells. Additionally, freely circulating viral proteins released from the virus contribute to the promotion of these virus-mediated complications. Based on an extensive literature review presented in this article, we propose that viruses promote the cellular production of ROS either directly by infecting host cells, or indirectly through the release of viral protein fragments in the blood circulation that elicit cell signaling. These ROS-dependent processes may contribute to the development of AD and other forms of dementia. Future studies are needed to provide the causal link between the roles of ROS and the development of neurological disorders associated with various types of viruses.

ROS is a common mechanism observed in both neurological disorders and viral infections. As discussed in this article, extensive research has emphasized the role of ROS in the pathogenesis of ADRD, as well as the cellular production of ROS by viruses associated with these conditions. However, surprisingly, the role of ROS in the relationship between viral infections and AD, as well as related dementias, remains underexplored. Future studies should focus on designing experiments to establish cause-and-effect relationships between specific ROS in neurological endpoints associated with ADRD caused by viruses such as SARS-CoV-2 and herpesviruses. Sophisticated antioxidant therapies cannot be ruled out as a potential means of reducing the burden of ADRD. Further research on the mechanism of ROS biology is crucial to accomplish such goals. In summary, we propose that viruses promote the cellular production of ROS either directly, by infecting host cells, or indirectly, through viral protein fragments in the blood circulation. This process may contribute to the development of AD and other forms of dementia (Figure 10).

In conclusion, by clarifying the interplay between viral infections, ROS biology, and neurodegeneration, we may uncover broadly applicable targets for diagnosis, prevention, and treatment of ADRD. This intersection of virology and neurodegeneration holds significant promise for reshaping our approach to age-related cognitive diseases.

## Figures and Tables

**Table 1:** Clinical Implications of Alzheimer’s Disease (AD) Progression.

Stage	Cognitive Symptoms	Behavioral/Psychiatric Symptoms	Functional Decline	Care Needs
**Early (Mild)**	-Memory lapses (names, words) -Difficulty with planning	-Mild anxiety or apathy	-Some difficulty with complex tasks	-Minimal assistance
**Middle (Moderate)**	-Disorientation -Trouble recognizing people -Language problems	-Agitation -Depression -Sleep disturbances	-Assistance with daily activities	-Supervision needed
**Late (Severe)**	-Severe memory loss -Inability to communicate	-Delusions -Aggression -Wandering	-Total dependence -Incontinence	-Full-time care or nursing home needs

**Table 2:** Clinical Biomarkers: Viral Infections, Oxidative Stress, & Neurodegeneration in Alzheimer’s Disease and Other Dementias.

Viral Infection	Biomarker	Sample	Pathophysiological Link	Diagnostic/Prognostic Value
**HSV-1, HSV-2**	Aβ42:40 ratio	CSF, plasma	Infection leads to APP processing toward Aβ42 & early amyloid pathology	Low ratio predicts conversion from MCI to AD & correlates with amyloid PET
**HSV-1**	pTau-181	CSF, plasma	HSV-1 reactivation increases tau phosphorylation via kinase activation & oxidative stress	Elevated pTau-181 = infection progression & correlates with neurofibrillary tangle burden
**HSV-1, CMV**	Neurofilament light chain (NfL)	CSF, plasma	Viral-induced axonal injury & neuroinflammation accelerate NfL release	Rising NfL levels predict neurodegeneration and cognitive decline
**HSV-1, CMV**	8-Hydroxy-2′-deoxyguanosine (8-OHdG)	CSF, plasma, urine	Oxidative DNA damage from viral-driven ROS overproduction	Elevated levels = oxidative stress & predict faster cognitive decline
**HSV-1, CMV, VZV**	F2-Isoprostanes	CSF, plasma	Lipid peroxidation from viral-triggered oxidative stress	Strongly associated with AD pathology & cognitive decline
**HSV-1, CMV**	Viral DNA (HSV-1 DNA, CMV DNA)	CSF, plasma	Direct detection of viral reactivation within CNS or periphery	Confirms active infection/reactivation; may guide antiviral therapy
**HSV-1**	Malondialdehyde (MDA)	Plasma, serum	ROS-induced lipid peroxidation linked to neuronal membrane damage	Higher levels = cognitive impairment severity
**HSV-1, CMV**	Glutathione Peroxidase (GPX)	Plasma, erythrocytes	Compensatory antioxidant enzyme activity during oxidative stress	Lower GPX = worse cognitive performance

## Data Availability

All data is contained within the article.

## Abbreviations

The following abbreviations are used in this manuscript:

Aβ	amyloid-beta
ACE2	angiotensin-converting enzyme 2
AD	Alzheimer’s disease
APOE-ε4	apolipoprotein E ε4
BHV-1	bovine herpesvirus 1
CMV	cytomegalovirus
CNS	central nervous system
COVID-19	coronavirus disease 2019
ETC	electron transport chain
H_2_O_2_	hydrogen peroxide
HIV	human immunodeficiency virus
HOCl	hypochlorous acid
HSV-1	herpes simplex virus 1
HSV-2	herpes simplex virus 2
KEAP1	Kelch-like ECH-related protein 1
MAPK	mitogen-activated protein kinases
NOS	nitric oxide synthase
NOX	NADPH oxidase
•NO	nitric oxide
Nrf2	nuclear factor erythroid-2–related factor 2
O_2_	molecular oxygen
•O_2_^−^	superoxide anion radical
•OH	hydroxyl radical
ONOO^−^	peroxynitrite
RNS	reactive nitrogen species
ROS	reactive oxygen species
SARS-CoV-2	severe acute respiratory syndrome coronavirus 2
SOD	superoxide dismutase
TLRs	toll-like receptors
VZV	varicella-zoster virus
